# TORC2 inhibition may boost DNA-damaging chemotherapy

**DOI:** 10.18632/oncotarget.3034

**Published:** 2014-12-30

**Authors:** Manuel Stucki

**Affiliations:** Department of Gynecology, University of Zurich, Schlieren, Switzerland

Certain types of DNA lesions activate a cellular signaling network that is usually referred to as the DNA damage response (DDR). The phosphatidyl-indsitol(3)- like kinases (PIKKs) ATM, ATR and DNA-PKcs are critical regulators of the mammalian DDR. ATM and DNA-PKcs are mainly activated in response to DNA double-strand breaks (DSBs) while the related kinase ATR is activated by a broader spectrum of DNA lesions including stalled DNA replication forks and DNA repair intermediates that contain long stretches of single-stranded DNA (ssDNA). Once activated, PIKKs phosphorylate a large number of downstream targets that are involved in the regulation of repair processes, the activation of cell cycle checkpoints and the triggering of apoptosis, should the damage be too severe to repair. Among the targets phosphorylated and activated by PIKKs are the two checkpoint kinases Chk1 and Chk2 that serve as signal transducer molecules. While Chk2 is downstream of the ATM signaling cascade, Chk1 is mainly activated through phosphorylation by ATR (reviewed in [1]).

The atypical Ser/Thr protein kinase target of rapamycin (TOR) also belongs to the PIKK family of kinases, but it has until recently not been implicated in the DDR. It mainly regulates nutrient-dependent signaling pathways underlying cell growth, proliferation and survival. TOR-dependent signaling pathways are often deregulated in cancer. TOR exists in two different complexes: TORC1 and TORC2. TORC1 primarily regulates growth and is involved in modulation of protein synthesis, ribosome biogenesis and autophagy. The cellular functions of TORC2 are less well understood, mainly because there is so far no specific inhibitor available for TORC2. In contrast to the starvation-like phenotypes observed upon disruption or inhibition of TORC1, loss of TORC2 generates diverse effects that often show species or cell type specificity (reviewed in [2]).

Recently, experiments carried out in yeast suggested a specific role of TORC2 in the maintenance of genome stability in response to the induction of DSBs and in response to oxidative or replicative stress [3,4]. Results by Selvarajah et al. [5] suggest that TORC2 is also implicated in the cellular response to DNA damage in mammalian cells. Specifically, it appears that TORC2 is required for the optimal phosphorylation and activation of Chk1 in response to treatment of cancer cell lines with Etoposide, a cytotoxic anticancer drug that causes DSBs. Failure to fully activate Chk1 in response to DNA damage usually results in defective cell cycle checkpoint activation and as a consequence, increased cell death. This is exactly what the authors observed: simultaneous treatment of several cancer cell lines with the TOR inhibitor PP242 and Etoposide led to abrogated cell cycle arrest and decreased survival when compared to treatment with Etoposide alone. While PP242 inhibits both TORC1 and TORC2, only depletion of the TORC2-specific scaffold protein Rictor had an effect on Chk1 phosphorylation, while depletion of the TORC1-specific subunit Raptor had no effect, thus indicating that the observed DDR phenotypes are specific for TORC2-dependent signaling events.

This raises the question as to how TORC2 regulates Chk1 phosphorylation in response to DNA-damaging chemotherapeutics. Surprisingly, at least in some cell lines, TORC2 inhibition not only led to a reduced Chk1 phosphorylation in response to Etoposide, but also to reduced Chk1 protein levels, probably as a result of reduced Chk1 translation. The reduced Chk1 expression appears to be specific to Etoposide treatment since ultraviolet light that also strongly activates the ATR-Chk1 route of the DDR did not decrease Chk1 protein levels upon TOR inhibition, even though Chk1 phosphorylation was still compromised.

Chk1 is a direct ATR target an in principle it is possible that the reduced Chk1 phosphorylation observed upon TORC2 inhibition is caused by a reduced ATR activation. Since efficient ATR activation depends on the generation ssDNA at sites of DNA lesion or blocked DNA replication forks, it will be worth exploring if ssDNA generation at sites of Etoposide-induced DNA damage requires TORC2 activity. In this context it is interesting to note that TORC2 inactivation was recently shown to increase the toxic effects of drugs that interfere with DNA replication in a mouse model for T-cell leukemia [6].

Is there any translational significance of these findings? Provided that TORC2 inhibition has few effects on its own, it may be worth exploring if TORC2 inhibition could sensitize tumor cells to chemotherapeutic drugs that cause DNA damage and/or interfere with DNA replication. Of course this would first require the successful development of a TORC2-specific inhibitor.
